# Application of Large Language Models in Stroke Rehabilitation Health Education: 2-Phase Study

**DOI:** 10.2196/73226

**Published:** 2025-07-22

**Authors:** Shiqi Qiang, Haitao Zhang, Yang Liao, Yue Zhang, Yanfen Gu, Yiyan Wang, Zehui Xu, Hui Shi, Nuo Han, Haiping Yu

**Affiliations:** 1 Shanghai East Hospital, School of Medicine, Tongji University, No. 1800 Yuntai Road, Shanghai, 200120, China, 86 18964538997; 2 Department of Emergency and Critical Care, Shanghai East Hospital, School of Medicine, Tongji University, Shanghai, China; 3 Neurological Rehabilitation Center, Shanghai Sunshine Rehabilitation Center, School of Medicine, Tongji University, Shanghai, China; 4 Department of Neurology, Shanghai East Hospital, School of Medicine, Tongji University, Shanghai, China; 5 Department of Gastrointestinal Endoscopy, Shanghai East Hospital, School of Medicine, Tongji University, Shanghai, China; 6 Department of Breast Diseases, Yueyang Hospital of Integrated Traditional Chinese and Western Medicine, Shanghai University of Traditional Chinese Medicine, Shanghai, China; 7 Department of Nursing, Zhongshan Hospital, Fudan University, Shanghai, China; 8 School of Acupuncture-Moxibustion and Tuina, Shanghai University of Traditional Chinese Medicine, Shanghai, China; 9 Department of Nursing, Shanghai East Hospital, School of Medicine, Tongji University, No. 1800 Yuntai Road, Shanghai, 200120, China, 86 18964538997

**Keywords:** large language models, stroke, artificial intelligence, home rehabilitation, health education.

## Abstract

**Background:**

Stroke is a leading cause of disability and death worldwide, with home-based rehabilitation playing a crucial role in improving patient prognosis and quality of life. Traditional health education often lacks precision, personalization, and accessibility. In contrast, large language models (LLMs) are gaining attention for their potential in medical health education, owing to their advanced natural language processing capabilities. However, the effectiveness of LLMs in home-based stroke rehabilitation remains uncertain.

**Objective:**

This study evaluates the effectiveness of 4 LLMs—ChatGPT-4, MedGo, Qwen, and ERNIE Bot—selected for their diversity in model type, clinical relevance, and accessibility at the time of study design in home-based stroke rehabilitation. The aim is to offer patients with stroke more precise and secure health education pathways while exploring the feasibility of using LLMs to guide health education.

**Methods:**

In the first phase of this study, a literature review and expert interviews identified 15 common questions and 2 clinical cases relevant to patients with stroke in home-based rehabilitation. These were input into 4 LLMs for simulated consultations. Six medical experts (2 clinicians, 2 nursing specialists, and 2 rehabilitation therapists) evaluated the LLM-generated responses using a Likert 5-point scale, assessing accuracy, completeness, readability, safety, and humanity. In the second phase, the top 2 performing models from phase 1 were selected. Thirty patients with stroke undergoing home-based rehabilitation were recruited. Each patient asked both models 3 questions, rated the responses using a satisfaction scale, and assessed readability, text length, and recommended reading age using a Chinese readability analysis tool. Data were analyzed using one-way ANOVA, post hoc Tukey Honestly Significant Difference tests, and paired *t* tests.

**Results:**

The results revealed significant differences across the 4 models in 5 dimensions: accuracy (*P*=.002), completeness (*P*<.001), readability (*P*=.04), safety (*P*=.007), and humanity (*P*<.001). ChatGPT-4 outperformed all models in each dimension, with scores for accuracy (mean 4.28, SD 0.84), completeness (mean 4.35, SD 0.75), readability (mean 4.28, SD 0.85), safety (mean 4.38, SD0.81), and user-friendliness (mean 4.65, SD 0.66). MedGo excelled in accuracy (mean 4.06, SD 0.78) and completeness (mean 4.06, SD 0.74). Qwen and ERNIE Bot scored significantly lower across all 5 dimensions than ChatGPT-4 and MedGo. ChatGPT-4 generated the longest responses (mean 1338.35, SD 236.03) and had the highest readability score (mean 12.88). In the second phase, ChatGPT-4 performed the best overall, while MedGo provided the clearest responses.

**Conclusions:**

LLMs, particularly ChatGPT-4 and MedGo, demonstrated promising performance in home-based stroke rehabilitation education. However, discrepancies between expert and patient evaluations highlight the need for improved alignment with patient comprehension and expectations. Enhancing clinical accuracy, readability, and oversight mechanisms will be essential for future real-world integration.

## Introduction

Stroke is a leading cause of mortality and disability among middle-aged and older adult individuals worldwide. According to the World Health Organization, more than 15 million people experience a stroke annually, resulting in approximately 6 million deaths and leaving millions more with varying degrees of long-term disability [1]. With the global aging population, stroke prevalence continues to rise, particularly in low-income and middle-income countries, where the disease burden is increasing, posing a significant public health challenge [2]. Stroke rehabilitation is critical for improving patient prognosis and quality of life [1]. Home-based rehabilitation, offering convenience and cost-effectiveness, has gained significant attention in recent years. However, successful home rehabilitation requires active patient engagement, effective family support, and professional guidance [3]. Comprehensive health education and rehabilitation instructions are essential to ensure that patients adhere to appropriate recovery protocols at home. Traditional health education methods, such as printed materials, books, and verbal instructions, often face challenges such as delayed information dissemination, inconsistent interpretation, and a lack of personalized support, which can hinder rehabilitation outcomes.

In recent years, large language models (LLMs) have rapidly advanced, gaining significant attention in the medical and health care fields due to their advancements in natural language processing. LLMs possess powerful language comprehension and generation capabilities, enabling them to deliver personalized, easily understandable health guidance tailored to patients’ needs [4,5]. Existing studies have explored LLM applications in patient education, mental health interventions, and health management [6,7]. However, LLMs vary considerably in terms of medical accuracy, comprehensiveness, safety, and readability.

To address these challenges, Shanghai East Hospital developed MedGo in 2024, a specialized Chinese medical LLM designed to assist health care professionals in clinical decision-making and medical consultations. MedGo has demonstrated exceptional capabilities in medical task processing, ranking among the top models in the Chinese Biomedical Language Understanding Evaluation benchmark and excelling in medical question-answering assessments [8].

To evaluate the practical effectiveness of LLMs in this context, we selected 4 representative models for comparison: ChatGPT-4, MedGo, Qwen, and ERNIE Bot. These models were chosen based on their diversity in orientation (general-purpose vs medical-specific), availability to Chinese users, and technological maturity at the time of study design. ChatGPT-4 and Qwen are general-purpose LLMs widely recognized for their advanced dialogue and multilingual capabilities. ERNIE Bot, developed by Baidu, is a leading domestic model with robust Chinese language processing. MedGo, as a medical-specific model developed within the Chinese clinical context, brings unique relevance to health care applications. This selection offers a balanced foundation for comparing the performance of LLMs in stroke rehabilitation health education.

The application of LLMs in the medical field is still in its early stages [9,10], with limited exploration of how to effectively integrate them into home rehabilitation for patients with stroke. Most existing studies [11] primarily focus on general-purpose LLMs (eg, ChatGPT and Google Bard), overlooking the potential of specialized medical models in health care [12]. Furthermore, comprehensive comparisons of various LLMs in medical applications are scarce.

This study aims to evaluate the effectiveness of multiple LLMs, including specialized medical models, in supporting home rehabilitation for patients with stroke. By offering a more precise and safer rehabilitation education pathway, this research seeks to advance the application of LLMs in health care, providing a more efficient and scientifically sound solution for home rehabilitation for patients with stroke. The findings have significant academic and practical implications for promoting the adoption of LLMs in health care.

## Methods

### Study Design

This study was conducted from January 5 to February 22, 2025. Using both quantitative and qualitative analyses, the study aimed to evaluate the effectiveness of LLMs in home-based stroke rehabilitation education.

### Study Subjects

This study compared 4 representative LLMs—ChatGPT-4, MedGo, Qwen-Max, and ERNIE Bot V3.5—based on their accessibility, medical relevance, and model diversity (Table 1).

**Table 1.** Study models.

Model	Version	Source	Medical-specific	Open source
Chat-GPT	V4.0	OpenAI (USA）	No	No
MedGo	—^a^	Laboratory of Biomedical Artificial Intelligence (China)	Yes	No
Qwen	Qwen-Max	Alibaba (China)	No	No
ERNIE Bot	V3.5	Baidu (China)	No	No

a Not available.

### Phase 1

#### Questionnaire Design

**Figure 1.**
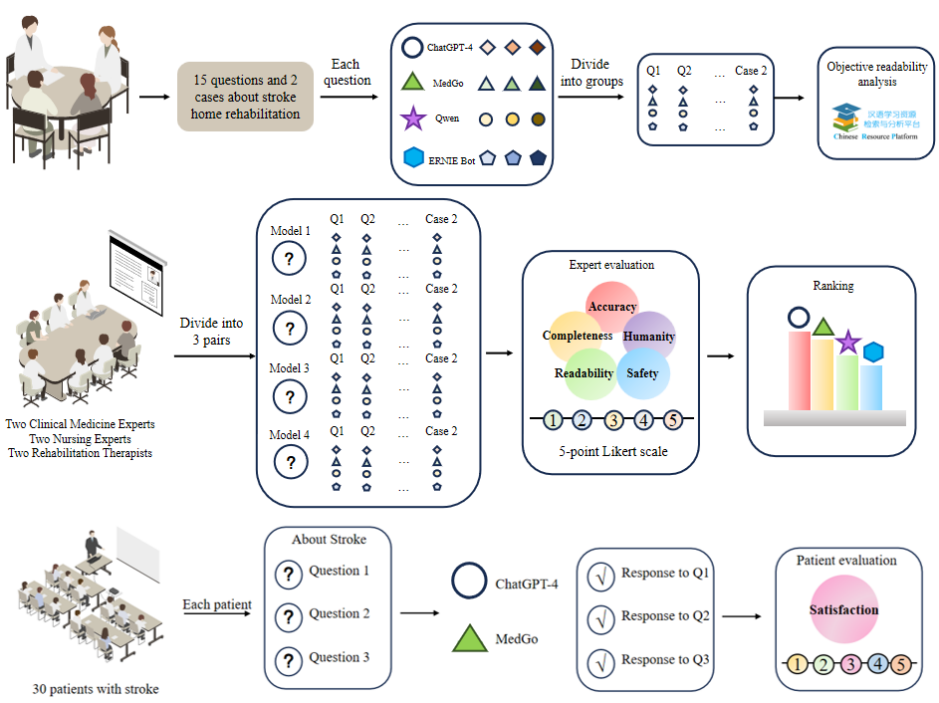
Research design workflow diagram.

The study aimed to evaluate the effectiveness of LLMs in home-based stroke rehabilitation education. By comparing the performance of responses from 4 different LLMs and incorporating expert ratings and patient feedback [13], we explored their practical application in the rehabilitation process. The detailed procedure is outlined in Figure 1.

Common inquiries about home rehabilitation for patients with stroke from the past 3 years were done and summarized from web-based medical platforms, including “DingXiangYuan,” “HaoDF,” “Baidu Health Ask a Doctor,” and “AliHealth.” Additionally, international stroke rehabilitation guidelines [14]—such as those from the American Heart Association, the American Stroke Association, and the Chinese Stroke Association—were reviewed to identify key concerns during the postoperative rehabilitation phase. Based on this information, an initial set of questions was selected.

Using this feedback and incorporating the clinical experience of neurorehabilitation nursing experts, a set of 15 targeted questions and 2 common home rehabilitation case scenarios were compiled. Case 1 focused on improving limb mobility and self-care abilities in daily life, with an emphasis on blood pressure control. Case 2 addressed not only limb and gait training but also speech and swallowing rehabilitation, diabetes management, and shoulder pain relief. The questionnaire is shown in Multimedia Appendix 1

#### Model Testing

To enhance the professionalism and specificity of LLM responses, ensuring that they address questions from a targeted perspective and align closely with real-world applications, a standardized prompt was added before each model-generated response. The prompt instructed the following:

*Assume you are an experienced rehabilitation specialist responsible for assessing the home rehabilitation needs of stroke patients and providing personalized advice based on their specific conditions. Use your professional knowledge to provide detailed answers to the following questions. Note that the inquirer is a patient or caregiver with no medical background. Ensure your responses include clear explanations and reference relevant medical evidence to aid understanding.*

Fifteen home rehabilitation questions and 2 clinical cases related to stroke were input into 4 different LLMs. Each model received the inputs 3 times, with each iteration conducted in a new conversation to eliminate prior chat history, allowing for an assessment of response consistency. The responses from all models were recorded in plain text format [15]. A single-blind method was applied: the 3 responses from each model were randomized and grouped into 4 sets of questions, which were then compiled into a questionnaire for expert evaluation.

#### Expert Evaluation

A Likert 5-point rating scale was used to assess the outputs of the 4 LLMs across five dimensions: accuracy, completeness, readability, safety, and humanity. (1) *Accuracy*: It evaluates whether the model-generated information aligns with scientific knowledge and medical facts. (2) *Completeness*: It assesses whether the model’s responses fully cover all relevant aspects of the question or case scenario. (3) *Readability*: It determines whether the model’s language is clear, concise, and easy to understand. (4) *Safety*: It examines whether the model provides safe and appropriate recommendations without potential risks. (5) *Humanity*: It assesses whether the model’s responses consider patients’ emotional needs, dignity, and individual differences while offering care and support.

Experts could provide brief explanations in the comment section if they had concerns about any response. The detailed definitions of the Likert 5-point rating scale are provided in Multimedia Appendix 1.

A total of 6 stroke rehabilitation experts (Table 2) were selected from tertiary general hospitals and specialized rehabilitation centers in Shanghai. The expert panel included 2 stroke center medical specialists, 2 neurorehabilitation nursing specialists, and 2 rehabilitation therapists, grouped into 3 teams [16]. This balanced composition ensured coverage of the key professional perspectives involved in home-based poststroke care while maintaining evaluation feasibility.

To minimize potential evaluator bias, all experts were blinded to the identities and affiliations of the 4 LLMs. The model outputs were anonymized and randomly ordered, with 3 independent rounds of scoring using distinct response samples in each round. Following a blind evaluation protocol, the experts assessed the outputs of the 4 LLMs across 5 dimensions. Prior to formal assessment, all experts attended a standardized training session and received a structured evaluation manual, which included detailed explanations of each scoring dimension, illustrative examples, and response interpretation guidelines. These measures aimed to promote consistent understanding of the evaluation framework and reduce interrater variability. Experts completed their assessments independently and were instructed not to communicate with one another during the scoring process. Additionally, the 6 experts conducted an interpretability analysis of the responses generated by the 4 LLMs. The expert scoring data can be found in Multimedia Appendix 2.

**Table 2.** Expert profiles.

Field of expertise	Expert	Degree	Title
Clinical medicine	Expert 1	PhD	Chief physician
Clinical medicine	Expert 2	PhD	Chief physician
Nursing	Expert 3	PhD	Chief nurse
Nursing	Expert 4	Bachelor’s	Associate chief nurse
Rehabilitation medicine	Expert 5	Master’s	Senior Therapist
Rehabilitation medicine	Expert 6	Master’s	Senior therapist

### Phase 2

Thirty patients with stroke were recruited in Shanghai. The 2 top-performing models from phase 1 were selected for interaction with the patients in a real clinical setting. Each patient asked both models 3 questions related to home-based stroke rehabilitation. The researchers recorded the responses and independently rated them using a satisfaction scale. Patient questions and scoring data can be found in Multimedia Appendix 3. See Textbox 1 for the inclusion and exclusion criteria.

Textbox 1. **Inclusion and exclusion criteria.**

**Inclusion criteria:**

- Patients diagnosed with stroke (including ischemic or hemorrhagic stroke) who meet the diagnostic criteria in the *2021 China Stroke Prevention and Treatment Guidelines* and have been confirmed by computed tomography or magnetic resonance imaging.
- Patients in the rehabilitation phase (1‐12 months post discharge, with ongoing rehabilitation needs).
- Patients undergoing home-based rehabilitation (not long-term hospitalized).
- Patients with basic communication skills, able to express their needs accurately (or with a family member to assist in communication).

**Exclusion criteria:**

- Patients with severe cognitive impairments or those unable to accurately express their needs.
- Patients unable to undergo home-based rehabilitation (eg, those requiring long-term hospitalization due to the severity of their condition).
- Patients with other serious comorbidities (eg, end-stage cancer and severe heart failure) that would interfere with the rehabilitation plan.

The assessment criterion was patient satisfaction, based on the following specific criteria (Textbox 2):

Textbox 2. **Patient satisfaction rating scale.**

Please select the option that best reflects your overall feeling toward the model’s response.

1 Point. Very dissatisfied: The response from the model is very unsatisfactory, completely failing to meet my needs.

2 Points. Dissatisfied: The response from the model is unsatisfactory, with many issues, and does not meet my needs.

3 Points. Neutral: The response from the model is acceptable; it answers some questions but still has room for improvement.

4 Points. Satisfied: The response from the model is satisfactory, and most of the questions have been answered effectively.

5 Points. Very satisfied: The response from the model is excellent, and all questions have been answered very well.

### Statistical Analysis

#### Primary Outcomes

Statistical analyses were performed using SPSS Statistics 27 (IBM Corp) and R (version 4.5.0; R Foundation for Statistical Computing). Descriptive statistics were used to summarize expert and patient ratings as means, SDs, and medians. In phase 1, differences among the 4 LLMs were assessed using one-way ANOVA with Tukey Honestly Significant Difference for post hoc comparisons if normality assumptions (tested via the Shapiro-Wilk test) were met. For nonnormally distributed or heteroscedastic data, the Kruskal-Wallis H test and Mann-Whitney *U* test with Bonferroni correction were applied. In phase 2, paired-sample *t* tests were used to compare patient ratings between 2 selected LLMs. To evaluate the consistency of expert ratings, interrater reliability was assessed using Krippendorff α for overall agreement and Cohen κ for pairwise agreement within expert subgroups (nursing, clinical, and rehabilitation). These analyses provided insights into potential variations in scoring standards across professional backgrounds.

#### Secondary Outcomes

An objective readability analysis was conducted on the responses generated by the 4 LLMs using a Chinese Readability Assessment Platform. This web-based tool [17,18] evaluates text readability by analyzing 52 linguistic features through a multiple linear regression model. The platform provides metrics, including education level, reading difficulty, and recommended reading age, with higher scores indicating more complex text.

A one-way ANOVA was used to assess differences among the 4 LLMs in terms of word count, reading difficulty scores, and recommended reading age. Post hoc analysis was performed using Tukey Honestly Significant Difference test to examine intermodel differences. Additionally, dot plots were generated using the HIPLOT web-based tool [19] to visually present the readability scores for each model. A significance level of α<.05 was adopted for all tests.

### Ethical Considerations

This study was approved by the medical ethics committee of Shanghai East Hospital (approval no.: 2025YS-042). All participants provided written informed consent prior to participation. Data collected from participants were anonymized to ensure privacy and confidentiality. Participants received a small gift (approximately RMB 30 [US $4.18]) as a token of appreciation for their time and involvement, in accordance with institutional ethics guidelines. No identifiable personal information is included in the manuscript or supplementary materials (Multimedia Appendix 4).

*Phase 1*: Common questions from patients with stroke undergoing home-based rehabilitation were collected, and based on the International Stroke Rehabilitation Guidelines and expert input, a questionnaire was developed containing 15 questions and 2 typical cases. These questions and cases were input into 4 LLMs—ChatGPT, MedGo, Qwen, and ERNIE Bot—with each model receiving the inputs 3 times. The models’ raw text responses were recorded. Two clinical medicine experts, 2 nursing specialists, and 2 rehabilitation therapists evaluated the responses using a Likert 5-point scale across 5 dimensions: accuracy, completeness, readability, safety, and humanity. This evaluation was conducted in 3 rounds, followed by statistical analysis of the ratings. *Phase 2*: Based on phase 1 results, the top 2 performing LLMs were selected for interaction with 30 patients, who provided satisfaction ratings [20].

## Results

### Phase 1: Primary Outcomes

Six experts evaluated the responses generated by the LLMs across 5 dimensions: accuracy, comprehensiveness, readability, safety, and user-centeredness. Table 3 shows the scores for each LLM.

Among the models, ChatGPT-4 achieved the highest scores across all dimensions, with particularly outstanding performance in safety (mean 4.38, SD 0.81) and humanity (mean 4.65, SD 0.65). MedGo performed well in accuracy (mean 4.06, SD 0.78) and completeness (mean 4.06, SD 0.74) but was slightly inferior to ChatGPT-4 in the humanity dimension. Qwen and ERNIE Bot received significantly lower scores than both ChatGPT-4 and MedGo. Table 3 visually shows the average score and highlights the overall performance trends among the models.

Descriptive statistics (Table 4) revealed significant differences across all five evaluation dimensions: (1) Accuracy: ChatGPT-4 achieved the highest score (mean 4.28, SD 0.84), followed by MedGo (mean 4.06, SD 0.78), while Qwen and ERNIE Bot both scored lower (mean 3.91, SD 0.77; mean 3.91, SD 0.81, respectively). (2) Completeness: ChatGPT-4 again led (mean 4.35, SD 0.75), followed by MedGo (mean 4.06, SD 0.74), with Qwen and ERNIE Bot receiving lower scores. (3) Readability: ChatGPT-4 scored the highest (mean 4.28, SD 0.85), followed by MedGo (mean 4.17, SD 0.81) and Qwen (mean 4.02, SD 0.81), while ERNIE Bot had the lowest score (mean 3.99, SD 0.79). (4) Safety: ChatGPT-4 topped this dimension (mean 4.38, SD 0.81), followed by MedGo (mean 4.23, SD 0.73), Qwen (mean 4.08, SD 0.78), and ERNIE Bot (mean 4.05, SD 0.75). (5) Humanity: ChatGPT-4 achieved the highest score (mean 4.65, SD 0.66), followed by MedGo (mean 4.38, SD 0.73), with Qwen and ERNIE Bot both scoring identically (mean 4.27, SD 0.72; mean 4.27, SD 0.75, respectively).

Among the 20 scoring files, Krippendorff α values ranged from 0.26 to 0.75. A total of 14 files (14/20, 70%) demonstrated at least fair interrater agreement (α≥.50). The remaining files (6/20, 30%) fell below the 0.50 threshold, indicating lower reliability. Dimensions such as safety and humanistic care generally showed higher consistency, particularly when evaluated for ChatGPT and MedGo. In contrast, comprehensiveness ratings and responses from Qwen and ERNIE Bot yielded more variability across expert scores. Cohen κ coefficients showed substantial variation across expert subgroups: clinical physicians had fair agreement (κ=0.28), while nursing experts (κ=0.03) and rehabilitation therapists (κ=−0.13) showed poor or even negative agreement (Table 5).

**Table 3.** Average scores of responses from 4 large language models and statistical significance of intermodel differences (*P* values).

Dimension	ChatGPT-4	MedGo	Qwen	ERNIE Bot	*P* value (vs MedGo)	*P* value (vs Qwen)	*P* value (vs ERNIE Bot)
Accuracy	4.28	4.06	3.91	3.91	—^a^	.005	.005
Completeness	4.35	4.06	3.90	3.96	.03	<.001	.002
Readability	4.28	4.17	4.02	3.99	—	—	.05
Safety	4.38	4.23	4.08	4.05	—	.03	.01
Humanity	4.65	4.38	4.27	4.27	.04	.001	.001

a Not available.

**Table 4.** Descriptive statistics of expert rating analysis and objective readability analysis (Chat-GPT4 and MedGo).

Dimension	Chat-GPT4		MedGo	
	Median (IQR)	Mean (SD)	Median (IQR)	Mean (SD)
Accuracy	5.0 (2.0-5.0)	4.28 (0.84)	4.0 (2.0‐5.0)	4.06 (0.78)
Completeness	5.0 (3.0‐5.0)	4.35 (0.75)	4.0 (2.0‐5.0)	4.06 (0.74)
Readability	5.0 (3.0‐5.0)	4.28 (0.85)	4.0 (2.0‐5.0)	4.17 (0.81)
Safety	5.0 (2.0‐5.0)	4.38 (0.81)	4.0 (3.0‐5.0)	4.23 (0.73)
Humanity	5.0 (3.0‐5.0)	4.65 (0.65)	5.0 (3.0‐5.0)	4.27 (0.73)
Chinese character count	1367.00 (916.00‐1697.00)	1338.35 (236.03)	998.00 (726.00‐1470.00)	1048.35 (195.26)
Reading difficulty score	12.81 (11.13‐15.16)	12.88 (0.82)	12.30 (11.21‐14.52)	12.38 (0.90)
Recommended reading age	13.00 (11.00‐15.00)	12.82 (0.78)	12.00 (11.00‐14.00)	12.29 (0.85)

**Table 5.** Results of expert consistency analysis (Krippendorff α).

Dimension	ChatGPT-4	MedGo	Qwen	ERNIE Bot
Accuracy	0.61	0.75	0.52	0.34
Completeness	0.56	0.52	0.54	0.26
Readability	0.68	0.75	0.58	0.54
Safety	0.75	0.70	0.59	0.43
Humanity	0.69	0.75	0.71	0.63

**Figure 2.**
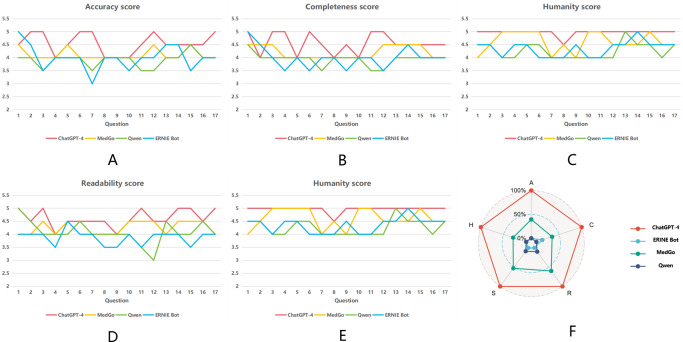
Line chart of the median scores and radar chart for the 4 large language models. (**A**) Accuracy, (**B**) completeness, (**C**) humanity, (**D**) readability, (**E**) safety, and (**F**) radar chart.

Figure 2 illustrates the performance of different LLMs across the 5 evaluation dimensions. Figures 2A-2E show the score trends for each model across 17 questions in terms of accuracy, completeness, humanity, readability, and safety, respectively. Figure 2F presents a radar chart comparing the overall normalized performance across all dimensions. ChatGPT-4 exhibited the best overall performance in all dimensions. The line chart shows the variation in average scores, with each line representing the scoring trend of a model across different questions. The radar chart offers a visual representation of each model’s performance across the 5 dimensions. Each axis of the radar chart corresponds to an evaluation dimension, with a larger area indicating stronger performance in that dimension.

### Phase 1: Secondary Outcomes

**Figure 3.**
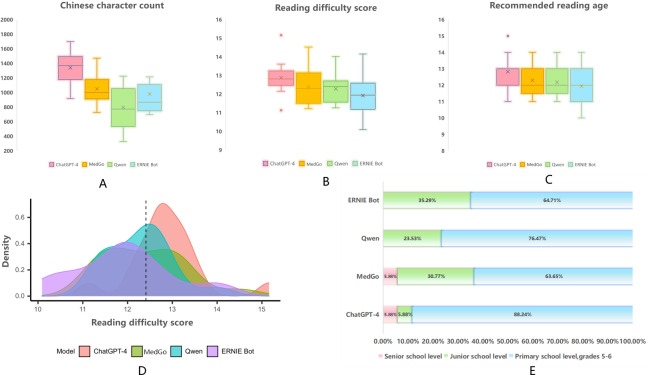
Comparative evaluation of large language model (LLM) responses on relevant questions. (**A**) Box plot showing the variation in text length among the 4 LLMs, with a significant difference observed between ChatGPT-4 and Qwen (*P*<.001). (**B**) Box plot illustrating the variation in reading difficulty scores among the 4 LLMs. (**C**) Box plot showing the variation in recommended reading age among the 4 LLMs (*P*=.07). (**D**) Density plot displaying the distribution of reading difficulty scores among the models. (**E**) Bar chart showing the distribution of educational levels required to comprehend the responses. *P* values indicate pairwise comparisons of Chinese character count: ChatGPT-4 versus MedGo (*P*=.002), ChatGPT-4 versus Qwen (*P*<.001), ChatGPT-4 versus ERNIE Bot (*P*=.02), and MedGo versus Qwen (*P*=.04); and for reading difficulty score: ChatGPT-4 versus MedGo (*P*=.01). All rating data in this study were tested and found to follow a normal or approximately normal distribution.

Descriptive statistics for the objective readability analysis are shown in Tables4  6 and Figure 3. Specifically, Figures 3A-3C display the variations in character count, reading difficulty, and recommended reading age across the 4 LLMs. Figure 3D shows the distribution of reading difficulty scores, and Figure 3E presents the proportions of education levels required to understand the responses. Chinese Character Count: ChatGPT-4 generated the highest total character count (22,752 characters) and the highest average word count (mean 1338.35, SD 236.03). In contrast, Qwen produced the shortest text, with a total of 13,481 characters and an average word count (mean 793.00, SD 283.64). Reading Difficulty Score: ChatGPT-4 had the highest average reading difficulty score (12.88), while ERNIE Bot had the lowest (11.92). Recommended Reading Age: ChatGPT-4 also had the highest mean recommended reading age (mean 12.82, SD 0.78 years), whereas ERNIE Bot had the lowest (mean 11.94, SD 1.43 years).

The results of the one-way ANOVA are shown in Table 7: Chinese Character Count: Significant differences were observed in the total text length among the LLMs (*F*_3,64_=11.43>*F*_crit_=2.75; *P*<.001), with the most significant difference between ChatGPT-4 and Qwen (*P*<.001). Reading Difficulty Score: A significant difference was found in reading difficulty scores among the models (*F*_3,64_=3.32>*F*_crit_=2.75; *P*=.03). Recommended Reading Age: No significant differences were observed in recommended reading age among the models (*F*_3,64_=2.48<*F*_crit_=2.75; *P*=.07).

**Table 6.** Descriptive statistics of expert rating analysis and objective readability analysis (Qwen and ERNIE Bot).

Dimension	Qwen		ERNIE Bot	
	Median (IQR)	Mean (SD)	Median (IQR)	Mean (SD)
Accuracy	4.0 (2.0-5.0)	3.91 (0.77)	4.0 (2.0‐5.0)	3.91 (0.81)
Completeness	4.0 (2.0‐5.0)	3.90 (0.78)	4.0 (2.0‐5.0)	3.96 (0.78)
Readability	4.0 (2.0‐5.0)	4.02 (0.81)	4.0 (2.0‐5.0)	3.99 (0.79)
Safety	4.0 (2.0‐5.0)	4.08 (0.80)	4.0 (2.0‐5.0)	4.05 (0.75)
Humanity	4.0 (2.0‐5.0)	4.27 (0.72)	4.0 (3.0‐5.0)	4.27 (0.75)
Chinese character count	772.00 (325.00‐1223.00)	793.00 (283.64)	867.00 (694.00‐2228.00)	979.12 (361.84)
Reading difficulty score	12.40 (11.26‐14.01)	12.28 (0.71)	11.95 (10.09‐14.15)	11.92 (1.09)
Recommended reading age	12.00 (11.00‐14.00)	12.18 (0.78)	12.00 (10.00‐14.00)	11.94 (1.43)

**Table 7.** One-way ANOVA of objective readability.

Dimension	*F* test (*df*)	*P* value	*F*_crit_
Accuracy	4.93 (3, 404)	.002	2.63
Completeness	7.01 (3, 404)	<.001	2.63
Readability	2.87 (3, 404)	.04	2.63
Safety	4.06 (3, 404)	.007	2.63
Humanity	6.18 (3, 404)	<.001	2.63
Chinese character count	11.43 (3, 64)	<.001	2.75
Reading difficulty score	3.32 (3, 64)	.03	2.75
Recommended reading age	2.48 (3, 64)	.07	2.75

### Phase 2: Patient Interaction Results

**Figure 4.**
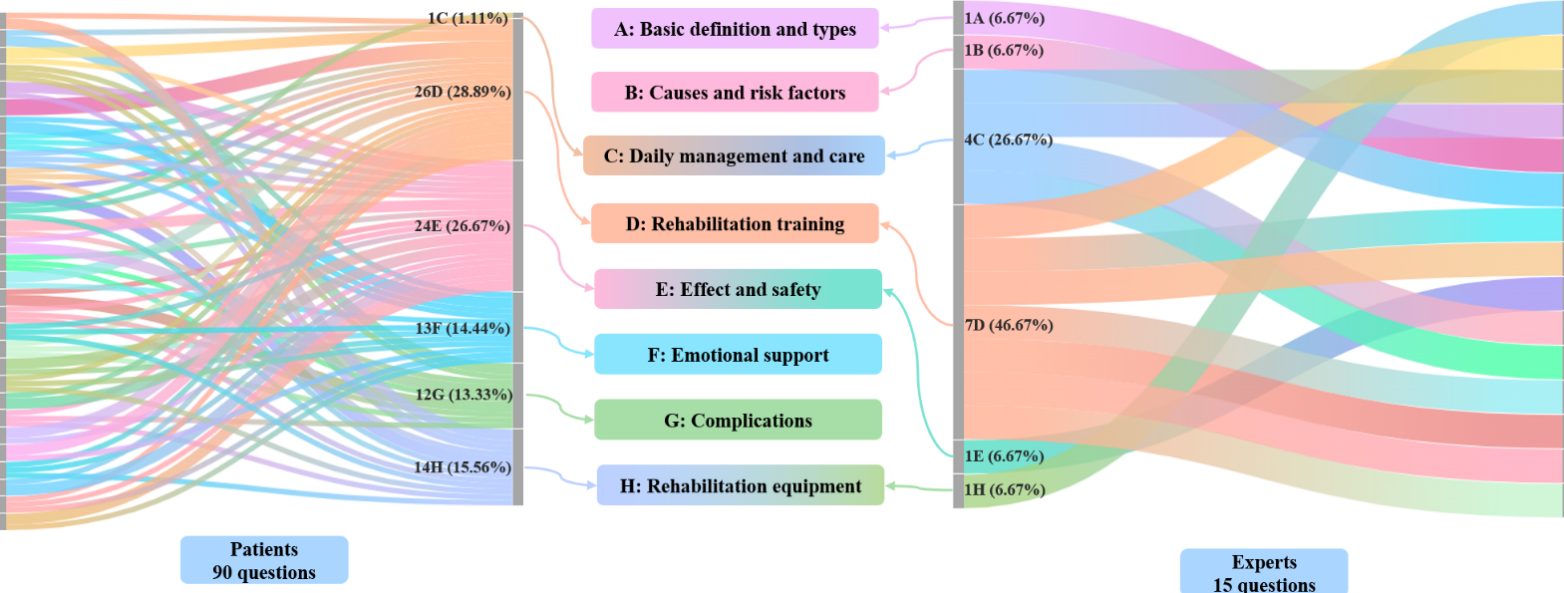
The Sankey diagram illustrates the classification of questions in both phases. On the left, 90 questions posed by 30 patients are shown, while on the right, the 15 integrated questions are displayed.

A total of 30 eligible patients were recruited, generating 90 questions (Figure 4). These, along with expert-suggested questions, were categorized into 8 groups: basic definitions and types, causes and risk factors, daily management and care, rehabilitation training, effectiveness and safety, emotional support, complications, and rehabilitation environment and equipment.

A 2-tailed paired *t* test showed that the average score for ChatGPT-4 (mean 3.34, SD  0.64) was slightly higher than that for MedGo (mean 3.04, SD  0.78), with a statistically significant difference between the 2 models (*t*_89_= 2.65; *P* = .01; 95% CI 0.08-0.53).

## Discussion

### Principal Results

This study evaluated the performance of ChatGPT-4, MedGo, Qwen, and ERNIE Bot in providing health education for patients with stroke undergoing home rehabilitation.

In phase 1, ChatGPT-4 demonstrated the best overall performance across all dimensions, excelling particularly in humanity and safety. This finding aligns with previous studies [21]. MedGo, as a medical-specific model, excelled in accuracy and completeness, underscoring its potential for medical text processing and generation. Qwen and ERNIE Bot received lower scores across all 5 dimensions than ChatGPT-4 and MedGo, indicating a significant performance gap. ChatGPT-4 showed a high and concentrated reading difficulty score distribution, making it well-suited for scenarios that require complex content generation. However, this may present readability challenges for general users. However, although its superiority in response quality was well demonstrated, ChatGPT-4’s high total character count and average word count may correlate with delayed response times. Prior studies indicated that LLM response latency increased by 0.8‐1.2 seconds per 100 Chinese characters on standard graphics processing units, which meant that ChatGPT-4’s responses required 10‐16 seconds to generate, which may impair the user engagement [22]. ERNIE Bot and MedGo exhibited lower and more stable readability scores, suggesting that they produce easier-to-read content, making them more suitable for general users or tasks that demand lower reading difficulty. Qwen displayed a wide range of readability scores, reflecting greater variability in reading difficulty but with relatively lower stability than the other models.

In phase 2 of patient interactions, ChatGPT-4 received higher ratings than MedGo, but overall, the ratings were lower than those given by the expert group. This discrepancy is mainly due to the challenges patients face in evaluating the models, including variations in personal understanding, needs, and the models’ performance and applicability. This is particularly evident when dealing with complex medical information. As supported by the inverse correlation between text length and satisfaction, ChatGPT-4’s detailed explanations and high reading difficulty could exceed the working memory and understanding capacity of the older adult, imposing heavy cognitive burden on them. Moreover, as artificial intelligence (AI) in medical decision-making is still developing, patients tend to be more skeptical of the models’ accuracy and reliability, often finding their responses unclear. In contrast, experts, with their accumulated knowledge and familiarity with medical terminology, are better equipped to interpret the models’ medical information, resulting in higher ratings.

There are significant differences in the areas of focus between patients and experts. Patients tend to prioritize rehabilitation methods, outcomes and safety, emotional support, and equipment-related concerns, reflecting their practical needs and psychological state during rehabilitation. Many patients with stroke are primarily concerned with improving their quality of life through daily management and care. Given the psychological pressures they face during rehabilitation, emotional support is also crucial. In contrast, experts focus on the effectiveness of rehabilitation plans, safety in technical aspects, and the dissemination of theoretical knowledge. As professionals, they are more likely to base treatment and rehabilitation plans on scientific evidence.

This disparity highlights the communication gap between experts and patients in health care. Patients may struggle to fully understand certain medical terms and treatment approaches, leading to confusion or anxiety about their rehabilitation plans. While experts emphasize treatment outcomes and safety, they must also consider how to effectively communicate this specialized knowledge to patients, fostering a correct understanding of rehabilitation and improving treatment adherence.

According to standardized prompts, each LLM was asked to provide sources for their responses. ChatGPT-4 did not explicitly cite references, but its answers, based on its extensive training dataset, generally aligned with medical knowledge and clinical practice. In contrast, MedGo provided more detailed medical support, citing specific medical literature, treatment guidelines, and clinical studies. However, the responses from Qwen and ERNIE Bot lacked clear citations of literature and concrete clinical evidence.

Some responses from the LLMs contained significant errors, which exposed critical safety vulnerabilities. For example, in question 3, which addressed the optimal period for stroke rehabilitation, Qwen incorrectly stated that the chronic phase of stroke begins 3 months after onset. According to various medical guidelines, the chronic phase typically starts 6 months after a stroke, making Qwen’s response inconsistent with these guidelines. Temporal misclassification may lead to premature termination of intensive therapy, reducing motor function recovery by 15%‐22% [23]. In question 7, concerning commonly used medications during home-based stroke rehabilitation, ERNIE Bot provided an incorrect answer, mentioning alteplase, a thrombolytic drug that cannot be taken at home and must be administered intravenously in a hospital setting, carrying a 6.7% risk of hemorrhagic complications if implemented [24]. The use of alteplase requires professional monitoring and must be administered within 4.5 hours of stroke onset. To improve this issue in future models, pretraining filtration should exclude non–hospital-administered medications from training data, while warnings ought to be triggered when responses contain high-risk medical terms. Furthermore, all medication-related queries need to be reviewed by clinicians afterward.

Analysis revealed that ChatGPT-4 made fewer errors, although it occasionally produced “hallucinations” due to issues with patient language expression. MedGo demonstrated high accuracy but lacked personalized care. Qwen and ERNIE Bot provided incomplete and vague responses.

The errors observed in the LLMs could impact patient rehabilitation to some extent. This is primarily because LLMs generate responses based on statistical language models rather than true understanding of the questions. They lack genuine comprehension and reasoning abilities. Their training depends heavily on large volumes of open-text data from the web, which does not guarantee the quality or timeliness of the answers. Medical knowledge is vast and complex, and the capabilities of LLMs vary. General-purpose LLMs struggle with specialized medical language and often lack explainability. These models are particularly prone to errors in areas such as disease diagnosis, drug effects, and emerging medical issues.

Therefore, when addressing medical questions, especially in health care decision-making, reliance on AI models should be approached with caution. Professional medical judgment remains irreplaceable, particularly when it concerns patient health and treatment plans.

### Comparison With Prior Work

A growing body of literature has evaluated the capabilities of LLMs across a wide range of clinical tasks, particularly in areas such as diagnosis support [25], test preparation [26,27], and medical documentation [28]. Most of these traditional LLM comparison studies involved models such as ChatGPT-3.5/4, Claude 2, Bard, and Qwen, and focused on measurable dimensions such as accuracy [29,30], specific competencies, and reasoning performance. These assessments often used standardized benchmark questions or national-level medical examinations as proxies for real-world expertise, such as the German Medical State Exam [31], the Chinese Nursing Licensing Exam [32], or the NEET-2023 in India [33].

In contrast, LLM applications in the domain of stroke and rehabilitation have been relatively limited and primarily focused on acute care or clinical prediction tasks. For example, one recent study used GPT-4 to predict 90-day mortality in ischemic stroke management [34] while another investigated the application of ChatGLM-6B in stroke diagnosis, subtype identification, and treatment eligibility screening [35]. These studies, although valuable, are oriented toward professional use. They focus on the performance of general-purpose LLMs [36,37], and research on medical-specific models remains limited. For instance, some studies have found that Med-PaLM 2 has made significant progress in medical question answering, particularly across multiple medical benchmarks and real-world problem-solving [38]. However, this model is not tailored for the Chinese medical context. Our study expands this line of inquiry by targeting a patient-facing use case—home-based stroke rehabilitation education—which involves not only clinical accuracy but also empathy, comprehensibility, and functional utility in patient self-management.

Furthermore, while ChatGPT-4 has consistently demonstrated strong performance in prior research [39], its superiority is not absolute. In our results, MedGo—a model developed for clinical Chinese QA—produced more concise and actionable responses in caregiver instruction tasks. This suggests that domain-adapted models may outperform generalist models when the prompt is tightly aligned with their specialization, echoing findings from recent studies in oral health and pediatric triage [40].

Another key distinction is our methodological framework. Many previous studies relied solely on expert judgments or automatic evaluation metrics. In contrast, our design includes both multidisciplinary expert ratings and real patient scoring, offering a dual-perspective validation framework. This approach provides ecological relevance by reflecting both clinical quality and user-perceived usefulness—dimensions often overlooked in LLM assessment [7].

Finally, our findings underscore the need for task-specific, longitudinal, and patient-centered evaluation of LLMs. Future research should incorporate more diverse rehabilitation populations, integrate multilingual models (eg, Qwen-2.5Max, Gemini, and Med-Gemini), and assess user trust, emotional alignment, and practical impact over time. The creation and evaluation standards for medical LLMs must be actively developed by the medical community [41] and validated through real-world experiments.

### Limitations

Although this study provides valuable insights into the application of LLMs in home-based stroke rehabilitation health education, several limitations should be noted. First, the expert sample size was small, with only 6 experts participating in the ratings, which may have affected the comprehensiveness and representativeness of the evaluations. The interrater consistency among experts was limited. This variability may stem from several factors, including differing professional perspectives, variable familiarity with LLM outputs, and inherent subjectivity in evaluating response quality across multiple dimensions. Furthermore, some rating criteria—such as “empathy” or “clinical applicability”—may be interpreted differently by clinicians and nonclinicians. Second, to facilitate patient understanding and streamline the rating process, only a satisfaction scale was used in the second phase of interaction, resulting in a simplified rating criterion. Third, the initial question design did not sufficiently address issues related to patient emotions and adherence. These dimensions were not apparent during phase 1, when questions were primarily developed based on medical platforms and existing literature—sources that tend to focus more on clinical procedures than on psychological or behavioral needs. However, during phase 2, through patient interviews and analysis of interaction data, we recognized that emotional fluctuations and treatment adherence play a vital role in the success of home-based rehabilitation. Fourth, the broader implementation of LLMs requires careful consideration of their economic viability, feasibility, and sustainability. While this study focused primarily on the academic perspective, real-world applications must address cost-effectiveness, technological barriers, and the potential impact on health care institutions. Based on comparable systems, deploying LLMs would incur application programming interface and graphics processing unit maintenance costs, while cost savings emerge when the clinic workload is reduced. However, whether breakeven can be approached remains unknown [42]. Fifth, model selection was constrained by temporal and practical considerations. Although ChatGPT-4.0, MedGo, Qwen-Max, and ERNIE Bot V3.5 were representative and widely used at the time of study design (January 2025), newer models such as Qwen-2.5Max and Med-Gemini were not included due to release timelines and limited accessibility. Specifically, Qwen-2.5Max was launched after the study protocol was finalized and required a paid subscription for application programming interface access, which may not reflect typical user access in community-based health care. Med-Gemini, although released earlier, had not been widely validated in peer-reviewed Chinese language medical research and remained restricted in practical application. In addition, domestic models such as Doubao (Cici) were excluded due to their lack of domain-specific medical optimization and low representation in scholarly health care literature. Finally, the health education content generated by LLMs still presents potential biases, inconsistent information, and a lack of explainability. Research [43] indicates that the use of LLMs in health care faces challenges related to information reliability, biases, ethical compliance, and patient acceptance.

Future research should focus on the following aspects. First, studies should consider increasing the number of expert raters within each professional subgroup to average out individual bias, standardizing scoring rubrics through iterative training, and using consensus-building techniques such as Delphi methods or calibration rounds prior to formal scoring. It may also be beneficial to incorporate mixed methods triangulation (eg, combining expert scores with patient evaluations or objective metrics) to strengthen the robustness of model performance assessments [44]. Second, a unified rating standard should be established when comparing expert and patient ratings to ensure result comparability. Specifically, for personalized home rehabilitation education for patients with stroke, future studies could expand the sample size and include evaluations from diverse patient groups, further exploring the adaptability of LLMs at different stages of rehabilitation. Third, subsequent research should consider participatory design strategies—such as structured patient interviews or patient-reported outcome measures—to ensure that emotional and motivational aspects are appropriately captured. Fourth, to address potential resource challenges in LLM application, feasibility assessments should be conducted regarding their management, use, and long-term maintenance. Fifth, research should expand the comparative framework to include newer LLMs such as Qwen-2.5Max, Med-Gemini, and international contenders such as Gemini [45], with a focus on evaluating their real-world performance in diverse clinical contexts. Longitudinal studies tracking LLM performance across updates may also be warranted to assess consistency, robustness, and scalability of health care–oriented outputs over time. Finally, further optimization of model algorithms is needed to improve the reliability of medical knowledge bases, and stronger oversight and regulation of AI-generated health information are essential [46].

### Conclusions

This 2-phase evaluation demonstrated that LLMs, particularly ChatGPT-4 and MedGo, show considerable promise in supporting home-based stroke rehabilitation education. ChatGPT-4 achieved the highest scores across all expert-evaluated dimensions, excelling in user-centeredness and comprehensiveness, while MedGo, a domain-specific model, produced more concise, evidence-based responses. In contrast, general purpose models such as Qwen and ERNIE Bot performed less consistently across key evaluation criteria. Notably, patients’ satisfaction ratings were lower than those of experts, highlighting potential usability challenges related to language complexity and trust in automated responses. These findings underscore the importance of aligning LLM-generated content with patient comprehension levels, emotional needs, and health literacy. Future work should focus on the development of hybrid models that integrate the conversational fluency of general purpose LLMs with the domain accuracy of medically trained models. Additional studies involving diverse patient populations, longitudinal designs, and real-world deployment scenarios are warranted to ensure safe, effective, and equitable integration of LLMs into patient-centered rehabilitation care.

## Supplementary material

10.2196/73226 Multimedia Appendix 1 Phase 1 questionnaire design and the detailed definitions of the Likert 5-point rating scale.

10.2196/73226 Multimedia Appendix 2 Phase 1 expert scoring data.

10.2196/73226 Multimedia Appendix 3 Phase 2 patient questions and scoring data.

10.2196/73226 Multimedia Appendix 4 Ethics committee approval document.

## Abbreviations

AI: artificial intelligence
LLM: large language model
