# Correction: Application of Large Language Models in Stroke Rehabilitation Health Education: 2-Phase Study

**DOI:** 10.2196/84717

**Published:** 2025-10-14

**Authors:** Shiqi Qiang, Haitao Zhang, Yang Liao, Yue Zhang, Yanfen Gu, Yiyan Wang, Zehui Xu, Hui Shi, Nuo Han, Haiping Yu

**Affiliations:** 1 Shanghai East Hospital, School of Medicine, Tongji University, Shanghai, China; 2 Department of Emergency and Critical Care, Shanghai East Hospital, School of Medicine, Tongji University, Shanghai, China; 3 Neurological Rehabilitation Center, Shanghai Sunshine Rehabilitation Center, School of Medicine, Tongji University, Shanghai, China; 4 Department of Neurology, Shanghai East Hospital, School of Medicine, Tongji University, Shanghai, China; 5 Department of Gastrointestinal Endoscopy, Shanghai East Hospital, School of Medicine, Tongji University, Shanghai, China; 6 Department of Breast Diseases, Yueyang Hospital of Integrated Traditional Chinese and Western Medicine, Shanghai University of Traditional Chinese Medicine, Shanghai, China; 7 Department of Nursing, Zhongshan Hospital, Fudan University, Shanghai, China; 8 School of Acupuncture-Moxibustion and Tuina, Shanghai University of Traditional Chinese Medicine, Shanghai, China; 9 Department of Nursing, Shanghai East Hospital, School of Medicine, Tongji University, No. 1800 Yuntai Road, Shanghai, 200120, China, 86 18964538997

In “Application of Large Language Models in Stroke Rehabilitation Health Education: 2-Phase Study” [1], the authors noted a few errors in the affiliations.

The authors’ information was originally published as follows:

Shiqi Qiang^1*^, MSc; Haitao Zhang^1,2*^, MSc; Yang Liao^1,3*^, MSc; Yue Zhang^1,4^, PhD; Yanfen Gu^1,5^, MSc; Yiyan Wang^1,3^, MSc; Zehui Xu^6^, BSc; Hui Shi^7^, MSc; Nuo Han^8^, BSc; Haiping Yu^1,9^, PhD

^*1*^ *School of Medicine, Tongji University, Shanghai, China*

^*2*^ *Department of Emergency and Critical Care, Shanghai East Hospital, Shanghai, China*

^*3*^ *Neurological Rehabilitation Center, Shanghai Sunshine Rehabilitation Center, Shanghai, China*

^*4*^ *Department of Neurology, Shanghai East Hospital, Shanghai, China*

^*5*^ *Department of Gastrointestinal Endoscopy, Shanghai East Hospital, Shanghai, China*

^*6*^ *Department of Breast Diseases, Yueyang Hospital of Integrated Traditional Chinese and Western Medicine, Shanghai University of Traditional Chinese Medicine, Shanghai, China*

^*7*^ *Department of Nursing, Zhongshan Hospital, Fudan University, Shanghai, China*

^*8*^ *School of Acupuncture-Moxibustion and Tuina, Shanghai University of Traditional Chinese Medicine, Shanghai, China*

^*9*^ *Department of Nursing, Shanghai East Hospital, Shanghai, China*

*these authors contributed equally

The information has now been corrected to:

*ShiqiQiang* ^1*^ *, MSc; Haitao Zhang* ^2*^ *, MSc; Yang Liao* ^3*^ *, MSc; Yue Zhang* ^*4*^ *, PhD; Yanfen Gu* ^*5*^ *, MSc; Yiyan Wang* ^*3*^ *, MSc; Zehui Xu* ^*6*^ *, BSc; Hui Shi* ^*7*^ *, MSc; Nuo Han* ^*8*^ *, BSc; Haiping Yu* ^*9*^ *, PhD*

^*1*^ *Shanghai East Hospital, School of Medicine, Tongji University, Shanghai, China*

^*2*^ *Department of Emergency and Critical Care, Shanghai East Hospital, School of Medicine, Tongji University, Shanghai, China*

^*3*^ *Neurological Rehabilitation Center, Shanghai Sunshine Rehabilitation Center, School of Medicine, Tongji University, Shanghai, China*

^*4*^ *Department of Neurology, Shanghai East Hospital, School of Medicine, Tongji University, Shanghai, China*

^*5*^ *Department of Gastrointestinal Endoscopy, Shanghai East Hospital, School of Medicine, Tongji University, Shanghai, China*

^*6*^ *Department of Breast Diseases, Yueyang Hospital of Integrated Traditional Chinese and Western Medicine, Shanghai University of Traditional Chinese Medicine, Shanghai, China*

^*7*^ *Department of Nursing, Zhongshan Hospital, Fudan University, Shanghai, China*

^*8*^ *School of Acupuncture-Moxibustion and Tuina, Shanghai University of Traditional Chinese Medicine, Shanghai, China*

^*9*^ *Department of Nursing, Shanghai East Hospital, School of Medicine, Tongji University, Shanghai, China*

*these authors contributed equally

The correction will appear in the online version of the paper on the JMIR Publications website together with the publication of this correction notice. Because this was made after submission to PubMed, PubMed Central, and other full-text repositories, the corrected article has also been resubmitted to those repositories.
