# Supplementary material for: Application of Large Language Models in Stroke Rehabilitation Health Education: 2-Phase Study
Source: J Med Internet Res. 2025 Jul 22;27:e73226. doi: 10.2196/73226 (PMC12306586; doi:10.2196/73226)
Supplement: Multimedia Appendix 1 [file jmir-v27-e73226-s001.docx]

Multimedia Appendix 1

Phase 1：Questionnaire Design

| Table S1. Questions and clinical scenarios. | |
| --- | --- |
| No. | Question |
| Q1 | How to recognize a stroke? |
| Q2 | How to prevent a stroke? |
| Q3 | What is the optimal period for stroke rehabilitation? |
| Q4 | How should stroke patients conduct home rehabilitation training after discharge? |
| Q5 | What special considerations should be taken into account during home rehabilitation for stroke patients? |
| Q6 | How should stroke patients and their families manage diet during rehabilitation? |
| Q7 | How should stroke patients manage medication during home rehabilitation? |
| Q8 | How should stroke patients use assistive devices for rehabilitation at home? |
| Q9 | How can the affected limbs of stroke patients be stimulated to promote recovery? |
| Q10 | What functions can patients regain through rehabilitation training? |
| Q11 | Does earlier rehabilitation lead to better outcomes? |
| Q12 | Can stroke patients fully recover through rehabilitation training? |
| Q13 | If no rehabilitation training is conducted, can patients still recover over time? |
| Q14 | Are individuals with a family history of stroke at higher risk of having a stroke? |
| Q15 | Do patients still need regular hospital check-ups during the home rehabilitation period? |
| Case1 | Mr. Shen, male, 71 years old, suffered a stroke two months ago and has been undergoing pharmacological treatment. The patient still experiences hemiparesis, with no active movement in the left upper limb and poor stability in the left upper and lower limbs, requiring assistance when walking. He also needs assistance with daily activities such as bathing. The patient has had hypertension for two years. How should he proceed with home rehabilitation? |
| Case2 | Mr. Wu, male, 77 years old, suffered a stroke one month ago and has been receiving pharmacological treatment. He still has residual weakness in the left side of his body, with impaired fine motor control in the left hand, an unstable gait when walking, difficulty with left-sided weight-bearing, and inability to stand independently. He also experiences frequent nighttime urination and needs assistance with eating, drinking, and bathing. After physical activity, he often feels weakness in his left lower limb. The patient has had hypertension for 40 years and was diagnosed with type 2 diabetes two months ago, with recent fluctuations in blood glucose levels. How should he undergo home rehabilitation? |

The detailed definitions of the Likert 5-point rating scale

| Table S2. Likert 5-point rating scale. | | | | | | |
| --- | --- | --- | --- | --- | --- | --- |
| Score | | Accuracy | Completeness | Readability | Safety | Humanity |
| 1 | The response is completely inaccurate, containing misleading or erroneous information. | | The response is extremely incomplete, missing key content. | The response is difficult to understand, with unclear and confusing language. | The response contains unsafe health recommendations or content that could mislead patients. | The response is very indifferent, ignoring the emotional needs of patients. |
| 2 | The response is partially accurate but contains some errors or incomplete information. | | The response is somewhat comprehensive but omits critical or important details. | The response is somewhat understandable but includes ambiguous or unclear parts. | The response is partially safe, but some suggestions may pose potential risks. | The response is somewhat indifferent, lacking empathy for patients. |
| 3 | The response is mostly accurate but contains minor errors. | | The response is mostly comprehensive, though some information is missing. | The response is mostly understandable, but some parts may require further clarification. | The response is largely safe, though certain suggestions may require further verification or revision. | The response is mostly human-centered but still has room for improvement. |
| 4 | The response is generally accurate, with few errors. | | The response is generally comprehensive, covering most relevant information. | The response is generally clear and easy to understand. | The response is generally safe and aligns with the patient's health needs. | The response demonstrates empathy and consideration for the patient's emotions. |
| 5 | The response is completely accurate, with no errors. | | The response is highly comprehensive, covering all relevant information. | The response is highly clear, concise, and well-structured. | The response is entirely safe, with no potential risks. | The response is highly human-centered, full of care and empathy. |
