# Supplementary material for: Application of Large Language Models in Stroke Rehabilitation Health Education: 2-Phase Study
Source: J Med Internet Res. 2025 Jul 22;27:e73226. doi: 10.2196/73226 (PMC12306586; doi:10.2196/73226)
Supplement: Multimedia Appendix 4 [file jmir-v27-e73226-s004.pdf]

上海市东方医院医学伦理委员会批件

Approval Letter of Ethics Committee, Shanghai East Hospital

批件号：【2025】研审第(042)号

Approval No: 2025YS-042

项目名称 Project	评估大语言模型在脑卒中患者居家康复中的应用				
申办方 Sponsor	上海市东方医院				
CRO	/				
类别 Classification	/	注册分类 Trial Objective	/	研究类型 Characteristics	观察性研究
专业名称 Department	护理	主要研究者 Principal Investigator	俞海萍	职称 Title	主任护师
审查方式 Review Method	☐会议审查 ☑快速审查			审查类别 Review Classification	初始审查
审查委员 Members	鲍欢、翟晓波				

审评材料 Documents	CFDA 批件/NMPA 通知书： ☐有 ☑否<br>检验报告： ☐有 ☑否<br>研究方案： ☑有 ☐否<br>病例报告表： ☐有 ☑否<br>知情同意书： ☑有 ☐否<br>招募广告： ☐有 ☑否<br>研究者手册： ☐有 ☑否<br>研究者资格： ☑符合 ☐不符合	主要参加单位 Participation	上海市东方医院
投票结果 Voting	会议时间：/<br>会议地点：/<br>其中：/		
审评意见 Comments	结论：☑同意 ☐作必要修改后同意 ☐作必要的修正后重审 ☐不同意 ☐暂停或终止已批准的试验<br>是否需要持续审查？ ☑是 ☐否 ｜ 持续审查频率：☐3 个月 ☐6 个月 ☑12 个月<br>审评意见：<br>经伦理委员会审议通过，审查结果为“同意”。<br>Tongji University School of Medicine, Shanghai East Hospital Ethics Committee<br>主任委员/副主任委员（签章）<br>Signature<br>日期：2025 年 02 月 13 日<br>Date of Issuance: 2025-02-13		

伦理委员会地址：上海市浦东新区即墨路 150 号　　邮政编码：200120

上海市浦东新区云台路 1800 号　　邮政编码：200123

伦理委员会联系电话：021-38804518-22157

CS 扫描全能王
3亿人都在用的扫描App

声明 Statement（请仔细阅读）：

1. 上海市东方医院医学伦理委员会（以下简称伦理委员会）遵循中国药物临床试验管理规范、医疗器械临床试验管理规范、ICH-GCP 和有关法规组成和工作，其审查工作过程不受伦理委员会以外任何组织及个人影响。
2. 本批件可能在其他中心机构及其伦理审查委员会备案。如对研究在贵机构实施的可行性（包括研究者的资格与经验、设备与条件等）有不同意见，请及时与本伦理审查委员会联系。
3. 研究实施前提：所有研究需经伦理委员会审查获得批件后方可实施，实施过程需遵循伦理委员会批准的方案执行，应符合 GCP 和《赫尔辛基宣言》的基本原则。特殊情况：①属《人类遗传资源采集、收集、买卖、出口、出境审批行政许可事项》规定范畴的研究，获得伦理批准后应按相关法规进行审批，获得中国人类遗传资源管理工作办公室批准后及时将批准文件交伦理委员会备案后方可实施。②属《需进行临床试验审批的第三类医疗器械目录》内医疗器械的临床试验，获得伦理批准后应按相关法规进行审批，获得 NMPA 批准后及时将批准文件交伦理委员会备案后方可实施。③如伦理审批前未获得 NMPA 批准文件的临床试验（含生物等效研究），请在获得备案回执或批准文件后及时递交伦理委员会备案，本伦理委员会备案后方可实施。
4. 研究过程中，对研究方案和知情同意书等相关文件所作的任何修订，均需得到伦理委员会审查同意后方可实施。
5. 本中心发生的严重不良事件或非预期不良事件，应按照相关法规要求报告本伦理委员会。
6. 暂停/提前终止临床研究，请及时通知本伦理委员会。
7. 方案违背和偏离须及时报告本伦理委员会。
8. 根据伦理审查委员会对持续审查频率的意见，无论试验开始与否，请在持续审查日到期前 1 个月提出持续审查的申请。
9. 完成临床研究，须提交结题报告供伦理审查委员会审查。
10. 本批件的有效期为 1 年，逾期未实施的则自动废止。

CS 扫描全能王
3亿人都在用的扫描App

上海市东方医院医学伦理委员会

审阅材料清单

Documents Submitted to Ethics Committee, Shanghai East Hospital

批件号：【2025】研审第(042)号

Approval No: 2025YS-042

审查材料 Documents Submitted（2025 年 02 月 13 日审查：同意）<br>1. 伦理审查申请表<br>2、研究方案（版本号：V1.0；版本日期：2025 年 02 月 05 日）<br>3、知情同意书（版本号：V1.0；版本日期：2025 年 02 月 05 日）

CS 扫描全能王
3亿人都在用的扫描App
